# Uterine Immunity and Microbiota: A Shifting Paradigm

**DOI:** 10.3389/fimmu.2019.02387

**Published:** 2019-10-17

**Authors:** Chiara Agostinis, Alessandro Mangogna, Fleur Bossi, Giuseppe Ricci, Uday Kishore, Roberta Bulla

**Affiliations:** ^1^Institute for Maternal and Child Health, IRCCS (Istituto di Ricovero e Cura a Carattere Scientifico) Burlo Garofolo, Trieste, Italy; ^2^Department of Life Sciences, University of Trieste, Trieste, Italy; ^3^Department of Medical, Surgical and Health Science, University of Trieste, Trieste, Italy; ^4^Biosciences, College of Health and Life Sciences, Brunel University London, Uxbridge, United Kingdom

**Keywords:** uterus, pregnancy, immune cells, cellular immunity, microbiota, menstruation

## Abstract

The female reproductive tract harbors distinct microbial communities, as in the vagina, cervical canal, uterus, and fallopian tubes. The nature of the vaginal microbiota is well-known; in contrast, the upper reproductive tract remains largely unexplored. Alteration in the uterine microbiota, which is dependent on the nutrients and hormones available to the uterus, is likely to play an important role in uterine-related diseases such as hysteromyoma, adenomyosis, and endometriosis. Uterine mucosa is an important tissue barrier whose main function is to offer protection against pathogens and other toxic factors, while maintaining a symbiotic relationship with commensal microbes. These characteristics are shared by all the mucosal tissues; however, the uterine mucosa is unique since it changes cyclically during the menstrual cycle as well as pregnancy. The immune system, besides its role in the defense process, plays crucial roles in reproduction as it ensures local immune tolerance to fetal/paternal antigens, trophoblast invasion, and vascular remodeling. The human endometrium contains a conspicuous number of immune cells, mainly Natural Killers (NK) cells, which are phenotypically distinct from peripheral cytotoxic NK, cells and macrophages. The endometrium also contains few lymphoid aggregates comprising B cell and CD8^+^ T cells. The number and the phenotype of these cells change during the menstrual cycle. It has become evident in recent years that the immune cell phenotype and function can be influenced by microbiota. Immune cells can sense the presence of microbes through their pattern recognition receptors, setting up host-microbe interaction. The microbiota exerts an appropriately controlled defense mechanism by competing for nutrients and mucosal space with pathogens. It has recently been considered that uterus is a non-sterile compartment since it seems to possess its own microbiota. There has been an increasing interest in characterizing the nature of microbial colonization within the uterus and its apparent impact on fertility and pregnancy. This review will examine the potential relationship between the uterine microbiota and the immune cells present in the local environment.

## Introduction

Mucosal barriers are the first line of immune defense designed to induce protection against noxious environmental agents including pathogens, and simultaneously allow symbiosis with commensal microbes (1). The largest number of commensals are segregated along the gastrointestinal tract; this segregation is achieved by the combined action of epithelial cells, mucus, IgA, anti-microbial peptides and immune cells (2, 3). One major benefit from the homeostatic relationship between the host and the microbiota is the resistance to pathogen colonization (4).

It has been demonstrated that the microbiota is able to influence the function of the host immune system, which keeps a symbiotic relationship with the microbiota. The ability of microbes to modulate the immunological response, both locally and systemically, requires sensing of microbes, followed by complex dialogue between innate and adaptive components of the immune system (2).

The uterine mucosal immune system is quite unique compared to other mucosal surfaces since it has to adapt to menstruation in response to hormonal stimuli (1). In addition to binding with the pathogens, the uterine immunity has evolved to tolerate the semi-allogeneic fetus, thus, playing a fundamental role in implantation and pregnancy (5).

The uterine microenvironment, for a long time, has been considered to be a sterile compartment, although the likely existence of commensal colonization within the uterus was always debated. Only recently, thanks to the discovery of the 16S rRNA in the uterine compartment, it has been possible to demonstrate and characterize the presence of commensal bacteria in the uterus. This review will focus on the immune system that characterizes the endometrium, the microbiota present in the uterine microenvironment, and their mutual homeostatic relationship.

## Anatomy of the Uterus

The female reproductive tract comprises the fallopian tubes, the uterus, and the vagina.

The uterus is the organ involved in the gestation; it has the function of accepting the fertilized egg and allowing its development. The uterus is an unequal organ, which at the top receives the outlet of the uterine tubes and at the bottom opens into the vaginal cavity (6–8).

The uterus, a pear-shaped viscus, can be divided into a fundus, body, and cervix. During pregnancy, it houses and supports the developing embryo and fetus. It is composed of a thick, muscular myometrium (covered by serosa and/or adventitia) and a spongy mucosal layer, the endometrium. The cervix is the inferior portion of the uterus, which protrudes into the vagina. The lumen (canal) of the cervix is continuous with the lumen of the uterus (superiorly) and the vaginal canal (inferiorly). It is divided into two main parts: the endocervix is the inner part of the cervix lining the canal leading into the uterus, whereas the ectocervix is the outer part of the cervix. It is rounded and lip-like and sticks out into the vagina. The endocervical canal is the passage from inside the uterus to the vagina (6–8). The superior surface of the uterus is convex and directed forward. The anterior surface is flat and looks downward and forward, resting on the bladder. Its peritoneal covering is reflected at the level of isthmus to the upper aspect of the bladder, creating the vesico-uterine pouch. The posterior surface of the uterus is convex and lies in relation to the pelvic colon and rectum. The peritoneum of the posterior wall covers the body and upper cervix, and then extends over the posterior fornix of the vagina to the rectum, to form the recto-uterine pouch or cul-de-sac of Douglas. The cervix is directed downward and backward to rest again the posterior wall. Only the upper half of its posterior surface is covered by peritoneum. The external side of the cervix lies at about the level of the upper border of the symphysis pubis in the plane of ischial spine (6–8).

The uterine wall is constituted by mucous or endometrium tunic, muscular or myometrium tunic, and serous tunic, where it is referred to as perimetry. A connective tissue constitutes the perimeter that surrounds the uterus, below the peritoneum, extending into the base of the broad ligament (6–8).

The endometrium, composed of an epithelial lamina propria, with its superficial functional and deep basal layers, undergoes hormonally modulated cyclic changes during the menstrual cycle. The three cyclic stages of the endometrium are the: follicular (proliferative) phase, luteal (secretory) phase, and the menstrual phase. The basal layer, which remains intact during menstruation, is served by short straight arteries and is occupied by the base of the uterine glands. The functional layer, served by the helicine (coiled) arteries, undergoes hormonally modulated cyclic changes. Follicle-stimulating hormone (FSH) facilitates the proliferative phase (follicular phase), characterized by thickening of the endometrium and the renewal of the connective tissue, glandular structures and blood vessels (helicine arteries) subsequent to the menstrual phase. Luteinizing hormone (LH) facilitates the secretory phase (luteal phase), characterized by the further thickening of the endometrium, coiling of the endometrial glands, accumulation of glandular secretions, and further coiling and lengthening of the helicine arteries. Decreased levels of LH and progesterone are responsible for the menstrual phase, which begins with long-term, intermittent vasoconstriction of the helicine arteries, with subsequent necrosis of the vessel walls as well as of the endometrial tissue of the functional layer. It should be noted that the basal layer is unaffected because it is supplied by the straight arteries. During relaxation (between episodes of vasoconstriction), the helicine arteries rupture, and the rapid blood flow dislodges the blood-filled necrotic functional layer, which becomes sloughed as the hemorrhagic discharge, so that only the basal layer of the endometrium remains as the lining of the uterus (7, 9).

## Characteristics of the Mucosa of the Female Reproductive Tract

As mentioned earlier, the female reproductive tract comprises the fallopian tubes, the uterus, and the vagina. The mucosa of the female reproductive tract differs between the upper and the lower tracts. The upper female reproductive tract that includes the uterine tubes, uterus and the endocervix, and is covered by a single-layered columnar epithelium. The lower reproductive tract includes the ectocervix and vagina, and is lined with a single layer of stratified, squamous, non-keratinized epithelium, which forms a more protective barrier than the columnar epithelium (10, 11).

Anti-microbial molecules, produced in the mucosa of the female reproductive tract, are significantly influenced by estrogen, which acts differently in the upper and lower reproductive tracts. High levels of estrogen, a characteristic of the pre-ovulatory period, increase the production of some anti-microbial peptides, such as secretory leukocyte peptidase inhibitor (SLPI), β-defensin 1-2 (HBD 1-2), and elafin from the endometrial epithelium (12). On the other hand, estrogen suppresses the LPS- and poly (I:C)-induced secretion of pro-inflammatory cytokines, including TNF-α, macrophage inflammatory protein 3α (MIP3α, CCL20), IL-1β, IL-6, and IL-8 from uterine epithelial cells. IgG levels in the uterine cavity peak in the periovulatory period. In contrast, the lower reproductive tract, including the cervix and the vagina, maintains a low level of IgA, IgG, and lactoferrin and decreased secretions of β-defensin 2, elafin, SLPI, and α-defensin1-3 (HNP1-3). Furthermore, concentrations of IL-6 and IL-8 in the vagina are decreased in the period of highest estrogen concentration (12). This decreased immunity in the lower genital tract may contribute to enhanced sperm survival in the periovulation period. In addition, the estrogen rise in the periovulatory period increases the levels of anti-microbial peptides and decreases pro-inflammatory cytokines in the upper genital tract, which prevents ascending infections by vaginal pathogens and provides favorable conditions for the transit of sperm and embryos.

## Uterine microbiota

The endometrium of healthy women, for a long time, has been considered as a sterile environment. However, the use of next-generation sequencing techniques has enhanced our understanding of the complexity and number of microbiota that inhabit human mucosal surfaces. Recently, it has been demonstrated that the sites of the body that were previously thought to be devoid of bacteria, such as the lungs (13), bladder (14), placenta and endometrium (15, 16), are in fact colonized by low-abundance and unique microbiota (17), each tissue and microenvironment hosting unique microbial communities (18).

The first study that provided information about uterine colonization by bacteria used endometrium of 78 patients after hysterectomy. The authors were able to show the presence of pathogens in culture in 6% of these patients. They also demonstrated the presence of carcinoma of the cervix but no other pathologic condition may predispose to microbial invasion of the uterine cavity (19).

The presence of microbes in the uterine cavity was later demonstrated by Moller et al. (20). A prospective study involved the uteri from 99 women admitted for hysterectomy for persistent irregular vaginal bleeding and fibromyomas. The uteri were opened under sterile conditions immediately after hysterectomy and samples were obtained from the isthmus and fundus of the uterine cavity for microbiological examination. In nearly a quarter of all the patients, the uterine cavity was colonized with potentially pathogenic organisms, harboring one or more microorganisms in the uterus, most notably *Gardnerella vaginalis, Enterobacter*, and *Streptococcus agalactiae*. Mitchell et al. recently evaluated the presence of vaginal bacterial species by species-specific quantitative PCR in excised uteri obtained from 58 patients (21). They detected mostly the presence of *Liners, Prevotella* spp. *and L crispatus* with the median quantities of bacteria 2–4 log_10_ lower those that present in the vagina. Recent studies via next generation sequencing have yielded important information about the presence of many genera of bacteria in the non-pregnant human uterus.

An interesting study by Moreno et al. analyzed endometrial fluid and vaginal aspirates obtained from 13 fertile women in pre-receptive and receptive phases within the same menstrual cycle (22). They also investigated endometrial fluid collected at pre-receptive and receptive phases from 22 fertile women to study the hormonal regulation of the endometrial microbiota. Genomic DNA was sequenced for the 16S ribosomal RNA (rRNA) gene. The data demonstrated that the presence of endometrial microbiota was not hormonally regulated during the acquisition of endometrial receptivity. Furthermore, the presence of a non-lactobacillus-dominated microbiota in a receptive endometrium was associated with significant decrease in implantation and pregnancy outcome.

Chen et al. analyzed the microbiota present in the lower vagina, posterior fornix, cervical mucus, endometrium, fallopian tubes, and peritoneal fluid obtained from the pouch of Douglas in women being operated for benign and non-infectious conditions (23). A significant fraction of the non-pregnant uterine microbiota comprises not only Lactobacillus spp., which is similar to the vaginal mucosa, but also a variety of bacteria that grow in mildly alkaline conditions (23). Although characterized by a lower bacterial biomass, endometrial samples have a higher bacterial diversity compared to the vaginal microbiota (24). The metabolic profiles of the vagino-uterine microbiota change throughout the menstrual cycle. The functions of these microbes in uterine mucosal immune homeostasis in non-pregnant and pregnant states remain to be ascertained (1).

Few studies have attempted to investigate the correlation between bacterial colonization and pregnancy outcome, by culturing catheter tips used for embryo transfer during *in vitro* fertilization. The results were not conclusive, probably due to the fact that in culture, the aerobic species dominate, in addition to potential contamination through the cervicovaginal canal (25).

## Immune Cells Residing in the Female Reproductive Tract

There are conflicting reports in terms of the leukocyte population in the female reproductive tract. This discrepancy arises from the differences in the sampling phase during the menstrual cycle, sample size, analytical methods, and antibodies used to identify immune cells (10). However, it is clear that the number of leukocytes per gram of endometrial tissue is greater than that of other reproductive tissues, including fallopian tube, endocervix, ectocervix, and vagina (10).

Immune cells residing in the reproductive tract are required to play paradoxical roles since they maintain immunity against pathogens and also establish immune tolerance for sperm and embryo/fetus in the upper tract (10). Natural killer (NK) cells and regulatory T (Treg) cells are extremely important in decidual angiogenesis, trophoblast migration, and immune tolerance during pregnancy (10). Dysregulation of endometrial/decidual immune cells is strongly associated with infertility, miscarriage, and other obstetric complications (10).

**Figure 1:**
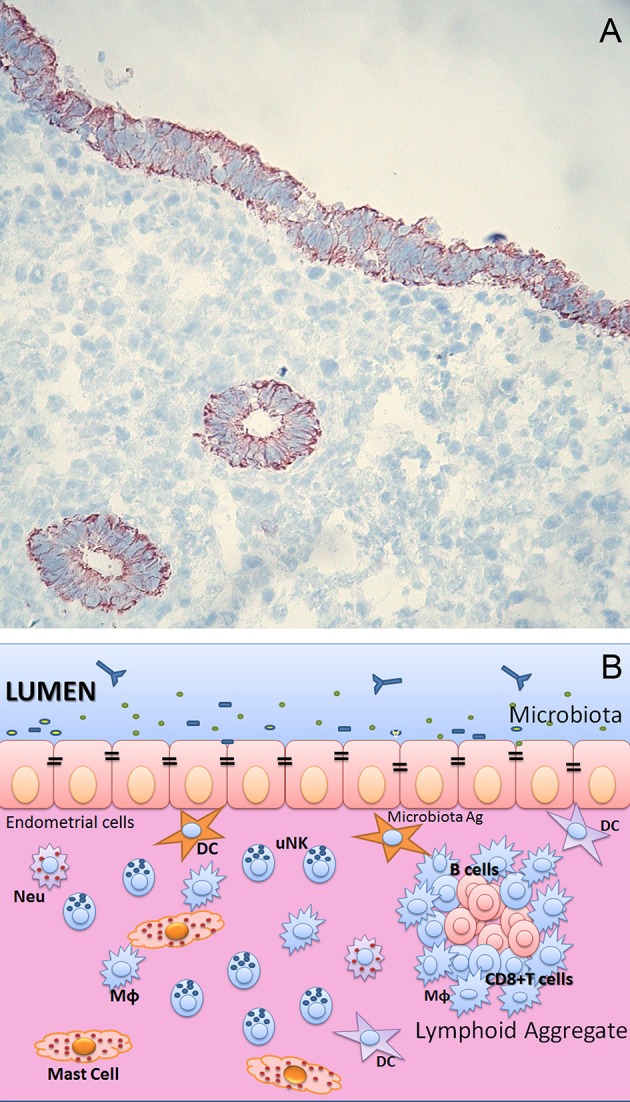
**(A)** Endometrium of human uterus. Immunohistochemical analysis of human endometrium on frozen section stained with CK7 monoclonal antibody (Dako) of uterus in proliferative phase. Bound antibody was revealed using the LSAB+ HRP kit and 3,3′-diaminobenzidine tetrahydrochloride (DAB) as chromogen. The sections were counterstained with hematoxylin. Monostratified columnar epithelium and the basal layer, which remains intact during menstruation, is occupied by the base of the uterine glands. Original magnifications 200×. **(B)** Immunological components of uterine mucosa. The endometrium is populated by a range of immune cells, such as mast cells, Macrophages (MΦ), Neutrophils (Neu), Dendritic cells (DC), T and B cells. The presence of lymphoid aggregates in the endometrial tissue suggests that this is an active site for cell-mediated immunity. Lymphoid aggregates found beneath the endometrium are composed of B cells in the inner core, surrounded by CD8^+^ CD4^−^ T cells and an outer layer of macrophages. Scattered CD56^+^ natural killer (NK) cells and CD4^+^ T cells can also be found in between lymphoid aggregates.

**Figure 2:**
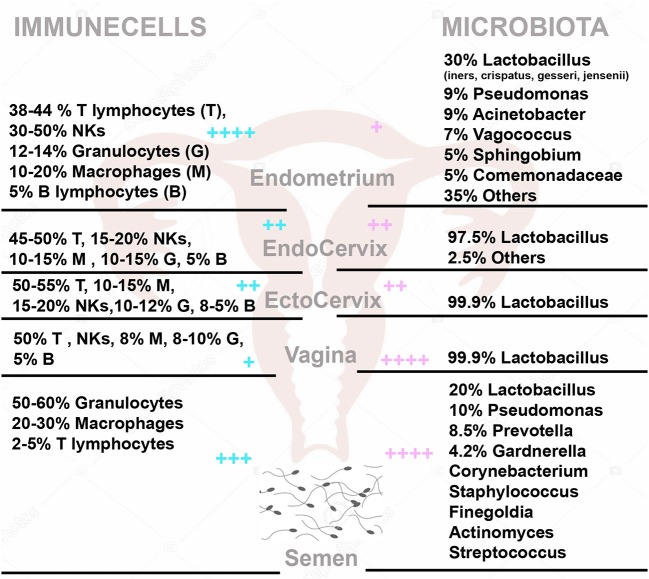
The immune cells **(Left)** and microbiota **(Right)** within the female genital tract and semen. On the left side are the percentage with (respect to total CD45 positive cells) of T Lymphocytes (T), NK cells (NKs), Granulocytes (G), Macrophages (M), and B Lymphocytes (B) in Endometrium, Endocervix, Ectocervix, Vagina and semen. On the right side, is shown the median relative abundance of microbial genera. + + ++/+ represent the relative abundance amount of microorganisms and immune cells in different areas.

The leukocytes in the female reproductive tract are distributed in either an aggregated or a dispersed form in the epithelial layer, lamina propria, and stroma (10). Although differentially distributed in each organ of the female reproductive tract, the predominant immune cells are T cells, macrophages/dendritic cells, NK cells, neutrophils, and mast cells (10). B cells are rare in the female reproductive tract (10) (Figures 1, 2).

Sex steroid hormones significantly influence these cells although they lack receptors for estrogen and progesterone (12, 26). As in the peripheral blood, leukocytes in the female reproductive tract also fluctuate in number cyclically through the menstrual cycle, probably as an indirect response to estrogenic and progesterone (12). The importance of these uterine immune cells is being discussed below.

### Innate Lymphoid Cells (ILCs)

Innate lymphoid cells (ILCs) are divided into three primary groups based on their phenotype and functions: type 1 (ILC1), type 2 (ILC2), and type 3 (ILC3) (27). Recently, the classification of ILCs has been be expanded to five subsets to reflect their distinct developmental pathways: NK cells, ILC1, ILC2, ILC3, and lymphoid tissue inducer (LTi) cells (28). It has been shown that only ILC3s are present in human endometrium and decidua, whereas all three groups of ILCs are present in the mouse uterus (29). The differences in the distribution of ILCs between human and mouse may reflect the dramatic changes that the mucosa undergoes in the human menstrual cycle with cyclical degeneration and renewal (29).

ILC1 include the prototypical NK cells and non-cytotoxic IFN-γ producing ILC1, characterized by expression of the transcription factor T-bet (27). ILC1s (Lin-CD56^+^CD127^−^CD117^−^RORγt^−^cells) are found in human non-pregnant endometrium, are further distinguished on the basis of their expression of NKp44 and CD103. CD103^+^NKp44^−^ cells are the principal source of IFN-γ (30), whereas, as previously described for tonsillar ILC1s (31), CD103^+^ expression facilitate the molecular communication between lymphocytes and epithelial cells (32), suggesting an epithelial association of such cells in the uterus.

ILC2 function through the release of helper T cell type 2 (Th2) cytokines such as IL-5 and IL-13 (33) and participate in immune responses, for example, against parasitic infection (34) and allergy (35); they also serve as systemic regulators of homeostasis (36). Most of the information about these cells are based on mouse models. For instance, murine uterine ILC2 are able to express the estrogen receptor α (37) and their percentage is increased in response to *in vitro* stimulation with 17β-estradiol.

ILC3 are divided into two main groups: LTi cells and non-LTi ILC3, referred to hereafter as ILC3 (27). For the first time, ILC3 have been described in the human non-pregnant endometrium. They represent a distinct subset of NK precursor-like cells that present ILC-associated markers such as CD127 and CD161 (38). The expression of the RORC and IL-22 genes that characterize an ILC3 phenotype, was also demonstrated (38). Subsequently, other studies confirmed the presence of ILC3 in the human endometrium (37, 38). These cells are divided into two main subsets: NCR^−^ (human NKp44^−^; mouse NKp46^−^) and NCR^+^ (human NKp44^+^; mouse NKp46^+^) ILC3s (7), with the NCR^−^ ILC3 being the dominant population in mice and the NCR^+^ ILC3 in humans. Apart from the NK cells, the potential role of other ILCs in the uterus is not fully known.

### Uterine NK Cells

Uterine NK cells have also been called as Large Granular Lymphocytes (LGL), endometrial granulocytes, K cells, endometrial Granulated Lymphocytes (eGL), and decidual Granulated Lymphocytes (dGL) (39). The major phenotype of endometrial NK cells is CD3^−^ CD56^bright^ CD16^−^, which distinguishes this cell subset from CD3^−^ CD56^dim^ CD16^+^ NK cells in the peripheral blood (10, 39). It is possible that peripheral blood CD56^bright^ NK cells home in to the uterus where they undergo tissue-specific differentiation. NK cells in the endometrium significantly expand in the late secretory phase and further increase their number during early pregnancy (10, 26).

In the proliferative phase, only a few NK cells are scattered throughout the stroma of the functional layer. However, they show a dramatic increase in their number after ovulation and continue to increase until a few days prior to menstruation. In the late secretory phase, the NK cells number surges up to 40% of the total cells in the stromal compartment and ~70% of endometrial leukocytes (10, 39). NK cells, particularly, surround both the arteries and glands of the endometrium. Since there are few NK cells in the endometrium in the menstrual and proliferative phases, an association between falling progesterone and apoptosis of uterine NK cells has been suggested. Considering that the NK cells and other leukocytes in the uterus do not express progesterone receptors, progesterone might exert its effects indirectly via cytokines or other soluble factors produced by stromal cells. Stromal cells strongly express both estrogen receptors (ERs) and progesterone receptors (PRs) (39). Carlino et al. demonstrated that uterine stromal cells from fertile or menopausal women were able to release chemerin, and exposure of uterine stromal cells to progesterone and 17β-estradiol resulted in enhanced chemerin release, supporting peripheral blood NK cell migration through uterine stromal cells (40).

### Macrophages

CD68^+^ macrophages are detected in all phases of the menstrual cycle (41). These cells also express specific markers such as CD71, CD69, and CD54 (42). These macrophages are scattered throughout the endometrium and are found especially around the glands (43). Tissue macrophages acquire distinct functional phenotypes, ranging between pro-inflammatory (M1) and anti-inflammatory pro-wound healing (M2) phenotype, in response to environmental cues (44). Studies using human tissues have revealed a progressive increase in the number of macrophages during the secretory phase of the cycle that peaks during menstruation, representing up to ~15% of the leukocyte population (45). CD68^+^ macrophages are abundant in the human endometrium during tissue breakdown and repair, and probably have a role in tissue clearance and tissue remodeling associated with menstruation (46). Macrophages within the endometrium have been suggested to have an important role in fertility and induction of a pro-inflammatory cytokine production (42). There is a significant increase in the macrophage number in the secretory phase, especially prior to menses, and this increase is particularly notable at the implantation site (39, 47).

### Neutrophils

Neutrophils, the most abundant leukocytes in the human immune system, comprise about ~10% of CD45^+^ cells in the cervix, with a greater presence in the ectocervix (11). Neutrophils have been identified in endometrial tissue by their morphology, by immunolocalization of the neutrophil-specific protease, elastase; in terms of surface markers, they have been defined as CD11b bright, CD66b^+^, and CD16^+^ (48). During most of the cycle, neutrophils are barely detectable in normal endometrium, but the numbers rise dramatically perimenstrually, making upto 6–15% of the total cell number in the tissue (41).

IFN-γ has also been detected in intra-epithelial neutrophils in human endometrium (48). In fact, neutrophils isolated from normal donors produce IFN-γ in response to stimulation with LPS, IL-12, and TNF-α. IFN-γ has a role in macrophage activation (49), suggesting at least one potential interaction between adjacent leukocytes during menstruation.

### Mast Cells

Mast cells are present in the endometrium throughout the menstrual cycle. They are detected by immunostaining for the two-mast cell-specific serine proteinases, tryptase, and chymase, and the extracellular relocation of the enzymes is indicative of their activation state (41).

Endometrial mast cell activation corresponds closely to the phases of tissue edema and is most marked prior to menstruation (50). As mentioned above, mast cells fall into two well-known phenotypic subtypes. Mucosal mast cells are positive for tryptase but not chymase, whereas connective tissue mast cells contain both enzymes. Both of these subtypes have been detected in endometrium, with regional differences. Those in the basalis region express both enzymes whereas those in the functionalis are positive only for tryptase (50). While it is the functionalis that is shed during menstruation, there is considerable tissue destruction at the basalis functionalis interface, and it is likely that both enzymes contribute to menstruation. Tryptase, and to a lesser extent, chymase, can play an important role in establishing a cascade of matrix metalloproteinase (MMP) activation and this could represent a critical function for these cells in the menstruation. Mast cells also produce histamine, heparin, arachidonate products and a variety of pleiotropic cytokines and growth factors, all of which have marked effects on endothelial cell function and local induction of edema (51).

### Dendritic Cells

Dendritic cells (DCs) are a heterogeneous and dynamic population of leukocytes; they are the most potent antigen capturing (immature DCs) and antigen presenting (mature DCs) cells. They are highly involved in the stimulation and modulation of the immune response within mucosal surfaces (52). In the basal layer of the endometrium, the density of endometrial CD1a^+^ immature DCs is significantly higher than that of CD83^+^ mature DCs throughout the menstrual cycle, whereas the number of CD83^+^ mature DCs remain relatively constant (39, 53). The number of CD1a^+^ immature DCs is likely to be indirectly regulated by steroid hormones (53). The density of CD83^+^ DCs was significantly greater in the basal layer compared with the functional layer during both the proliferative and secretory phases, whereas for CD1a^+^ DCs, the greater density in the basal layer was only observed in the secretory phase (53). Cyclical changes in DC populations during the normal menstrual cycle may be important for local regulatory mechanisms relevant to menstruation and implantation. Alterations in this normal profile may contribute to menstrual disturbances and fertility.

### Lymphocytes

CD3^+^ T cells are a minor population in the endometrium, comprising only ~1–2% of the total lymphomyeloid cells (45). These T cells are distributed at three different sites of the endometrium: aggregated in the basal lymphoid, and scattered in the stroma, and in the epithelial sites (41). In contrast to CD3^+^ T cells in peripheral blood, endometrial CD3^+^ T cells consist of a larger proportion of CD8^+^ cells (66%) and smaller proportion of CD4^+^ cells (33%) (41). Cytolytic activity of endometrial CD8^+^ T cells is maintained during the proliferative phase, but this activity weakens in the secretory phase without any drop in the CD8^+^ cell number (12, 41). This suppression of CD8^+^ cytolytic activity has been observed only in the fallopian tubes and endometrium, but not in the cervix and vagina (12). CD45RA^+^ T cells were found throughout the cycle, but their numbers are very low (41).

Hormonal changes may be drivers for Treg changes. In particular, estrogen has been shown to induce expansion of Foxp3^+^ Treg cells (54, 55), including in the pregnant uterus (56), which is essential for promoting immune tolerance toward the fetus. Activation of Treg is needed for a successful pregnancy, while suppression of Treg was associated with pregnancy failure (56).

### Gamma/delta (γδ) T Cells

Two types of T cells, αβ T cells and γδ T cells are present in vertebrates and are defined by surface expression of either the αβ or the γδ TCR complex. Thymus is the origin tissue of both these subsets but there are several key differences between αβ and γδ T cells: (i) αβ T cells are primarily localized in secondary lymphoid organs, whereas γδ T cells are predominant at epithelial surfaces; (ii) αβ T cells recognize peptide ligands in the context of MHC class I and class II molecules, whereas γδ T cells do not recognize peptides in an MHC-restricted manner but recognize and respond to a broad range of antigens, including heat shock proteins, and lipids; and (iii) the role of αβ T cells in immune response is well-defined whereas that of γδ T cell are not yet fully characterized (57).

Haller et al. analyzed the presence of γδ-positive cells in non-pregnant endometrium, and in first trimester and third trimester basal decidua. They did not observe any substantial differences in the number of γδ T cells in first and third trimester basal decidua compared with the non-pregnant endometrium. γδ T cells were occasionally found scattered throughout the endometrial/decidual stroma and showed no clear distribution pattern, nor was there any obvious relation to the pregnancy in the first or third trimester (58). Although Heyborn et al. showed that γδ T cells were significantly enriched at the feto-maternal interface, compared to the periphery or the non-pregnant uterus (59), the role of these lymphocytes in pregnancy is not yet fully understood. Hill et al. observed an increased number of TcRγδ-positive cells in spontaneous miscarriages endometrium compared to normal pregnancies (60). Vassiliadou and Bulmer showed that γδ-T cells were present in both normal pregnancy and spontaneous pregnancy loss, often located adjacent to endometrial glands (61). These cells formed a very small proportion of T cells in both groups and there was no difference in their numbers and proportions between normal and pathological tissues.

## Endometrial Lymphoid Aggregates

Endometrial lymphoid aggregates comprise a CD19^+^ B cell core, surrounding T cells, and an outer halo of macrophages in the stratum basalis (12, 39). These lymphocyte aggregates vary in size with the phase of the menstrual cycle, becoming larger in the secretory phase (3,000~4,000 cells) than the proliferative phase (300~400 cells) (12). To form lymphoid aggregates, immune cell trafficking to the nucleation sites is likely to be the principle mechanism, rather than *in situ* proliferation of precursor cells (62). Recruited immune cells from the circulation are likely to undergo tissue-specific differentiation in the local microenvironment, which confers new characteristics on these tissue-specific cells that are distinct from their original properties (39, 63).

## Cyclic Fluctuations of Endometrial Leukocytes

**Figure 3:**
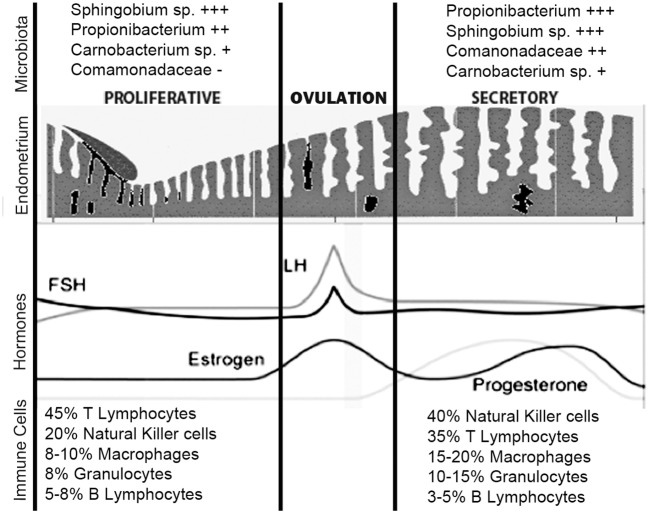
Uterine immune stages during menstruation cycle. During the normal menstrual cycle, the human endometrium is exposed to cyclical fluctuations of sex hormones. The repetitive cycles of proliferation, differentiation, decidualization, and shedding of this tissue during mensuration and the steroid hormones *per se* cause profound changes in the immune cells' population. The estrogen-dominant proliferative phase is characterized by the regeneration of the functional layer of the endometrium. In the progesterone driven secretory phase, the endometrium undergoes a number of changes in preparation for implantation of the embryo. The immune cells that undergo greater number variations in the secretory phase are the NKs and macrophages. The microbiota, on the other hand, does not undergo large variations during different phases of the menstrual cycle. *Sphingobium* sp., *Propionibacterium* acnes, and *Pseudomonas* sp. are differentially enriched during the proliferative and secretory phases; *P. acnes* is more abundant in the secretory phase and has previously been identified in the placenta and cultured from follicular fluid. Functionally, the proliferative phase, compared to the secretory phase, appears associated with increased bacterial proliferation. + + ++/+ represent the relative abundance amount of microorganisms.

Endometrial leukocytes exhibit profound cyclic fluctuations between the follicular and luteal phases of the menstrual cycle (10). The mean number of CD45^+^ cells in the endometrium remains low from the early follicular phase to the early secretory phase, but increases considerably (about 5-fold) during the secretory phase (64). The major population of CD45^+^ leukocytes in the late secretory phase is NK cells comprising ~80% of CD45^+^ cells, while CD3^+^ T cells decrease to <10% (64). Even though the percentage of CD3^+^ T cells is lower in the late secretory phase, the absolute number of T cells remained unchanged during the menstrual cycle. Comparing the late proliferative phase with the late secretory phase, the proportion of CD3^+^ CD8^+^ T cells decreases significantly from 63 to 54% (64) (Figures 2, 3).

The number of CD3^−^ CD56^+^ NK cells significantly increases in the late secretory phase compared with other phases (64). The cytotoxicity of endometrial NK cells is high, comparable to peripheral NK cells in the late proliferative phase (65). Similarly, the expression of the activation markers, CD69 and HLA-DR, is increased on NK cells in the proliferative phase (66). These characteristics of NK cells are likely to contribute to protective immunity against microbial infections.

During the menstrual cycle, neutrophils and eosinophils remain at a very low number in the endometrium, but their numbers profoundly increase in the premenstrual period, up to 15% and 5% of endometrial cells, respectively (41, 45).

CD68^+^ macrophages were found throughout the cycle and significantly increased from the proliferative phase to the secretory phase (67). The density of endometrial CD1a^+^ DCs, but not CD83^+^ DCs, in the basal layer gradually increased through the menstrual cycle, showing a nadir at the proliferative phase and reaching its peak in the menstrual phase (53). Only in the secretory phase was the density of CD1a^+^ DCs greater in the basal layer than in the functional layer. Endometrial CD83^+^ DCs showed greater density in the basal layer than in the functional layer throughout the menstrual cycle (53).

## Role of Cervical Mucus in Bacterial Uterine Colonization

The portal between the uterus and the vagina is represented by the cervix, which functions as an entrance to the endometrial cavities. Cervix contains several hundred crypts (glands) lined by cells which, under hormonal and neural influence, produce mucus, composed mostly of water (95–99%) and a complex mixture of organic components, inorganic ions, enzymes, mucins, and a high concentration of cytokines, anti-microbial peptides, immunoglobulins, and MMPs to protect the uterus against bacterial colonization (68, 69). Mucus released into the cervical canal moves into the vagina, where it acts as pathogen traps. It is an important protective barrier, preventing the ascendance of microorganisms into the uterus cavity; on the other hand, it is essential for the migration of spermatozoa. It has been shown that the hydration of the mucus and its glycosylation state play a pivotal role in both these processes (70, 71). Mucins in the cervix are known to change conformation during the menstrual cycle due to pH; these variations possibly allow passage of bacteria from vagina (72). However, bacteria from the lower female reproductive tract are able to cross. Hansen et al. have shown that the cervical mucus plug only inhibits (and does not completely block) the passage of *Ureaplasma parvum* during its ascendance from the vagina through the cervical canal (17, 73).

## Role of Semen in Bacterial Uterine Colonization

Besides all the putative bacterial transmission routes between uterine microbiota and distal sites such as hematogenous spread of bacteria through either oral or gut route, migration of bacteria through the cervix, and retrograde spread through fallopian tubes, another possible transmission route that influences uterine microbiota is the semen (24, 69). It is essentially composed of the spermatozoa cells and seminal plasma, which contains many components contributing to the immune tolerance at foeto-maternal interface (74). Semen, despite being synthesized in a tissue that is not typically colonized by a microbiota, is very far from being sterile; even in normal individuals, usually the microbial count is 10^3^ organisms/mL semen (75). Weng et al. showed that the most abundant genera present in seminal fluid are *Lactobacillus* (19.9%), *Pseudomonas* (9.85%), *Prevotella* (8.51%), and *Gardnerella* (4.21%) (Figure 2). Unsupervised clustering analysis revealed that the seminal bacterial communities were clustered into three main groups: *Lactobacillus, Pseudomonas*, and *Prevotella* predominant group (76). For this reason, the seminal fluid is a microorganism source for female genital tract. Indeed, it has been shown that bacteria are shared among partners influencing the species composition of each other's reproductive tract microbiota (24). Semen, being slightly basic and enriched with carbohydrates, represents an ideal habitat for microorganisms so that it may function as a perfect medium for the transmission of microorganisms, which finally may reach the uterus and become resident.

## Perspectives

Mucosal barriers are the first line of immune defense against the external environment and one major benefit resulting from the homeostatic relationship between the host with the commensal microbiota is the resistance to pathogen colonization (4). Although the uterine microenvironment has been considered a sterile compartment in the past, it is now clear that some commensal bacteria are present in the uterus. Therefore, it is possible to hypothesize a cross-regulation of the uterine microbiota and the local immune system. The uterine microbiota and the uterine immune system have to adapt to the functional changes of the uterus: first of all, they have to adapt to the cyclic hormonal stimulation during the menstrual cycle implantation and pregnancy. The uterine microbiota and the uterine immune cells must together tolerate the paternal antigens in response to the immunological stimulation by the semen, the embryo and the fetus.

We have provided an overview of the immunological order in an healthy endometrium since the recognition of the changes in the local microbial communities may point toward reproductive failure at different levels (from implantation failure in the uterus to the pregnancy complications), as well as other gynecological diseases (10, 11, 77). Furthermore, alteration of the uterine microbiota can also be a potential trigger for tumorigenesis. It is well-known that the etiology and progression of cancer may be influenced by microbial infections, as is *Helicobacter pylori* for gastric cancer, and *Fusobacteria* and *Porphyromonas* for colorectal cancer. It has been suggested that the presence of *Atopobium vaginae* and *Porphyromonas* sp. in the gynecologic tract is statistically associated with endometrial cancer (78).

It is an emerging notion that the microbiota is able to influence the function of the host immune system, which keeps a symbiotic relationship with the microbiota. Normally in other mucosa, such as the intestinal mucosa, bacteria are confined to the luminal side of the epithelial mucosa but occasionally they breach the physical barrier. These commensal microbes that penetrate the lamina propria are phagocytosed by resident macrophages and engulfed and carried by DCs to the local lymphoid aggregates and consequently modulate the adaptive immune system (79). The nature of interplay between uterine microbiota and the immune cells is not known. One can only speculate that a similar mechanism may work in the case of uterine compartment involving local lymphoid aggregates. The symbiotic relationship between the uterine microbiota and the innate and adaptive immune system probably has a fundamental role in maintaining a balanced inflammatory milieu since this mild bacterial stimulation could induce a potentially favorable microenvironment for embryo implantation. On the other hand, regulated stimulation of the immune system by microbiota could facilitate induction of tolerance to the non-sterile semen traversing through the uterine cavity.

In conclusion, we have discussed the potential role of microbiota in the control of uterine cells present in the microenvironment. We deliberately avoided reviewing and summarizing the literature on the uterine microbiome in animal models because our aim was to focus on human milieu. This can be a limitation since important information on local mechanisms can be obtained only using animal models. For example, it is possible to compare the reproductive performances of germ free and conventional animals and to study the rates of “immune abortion” in germ free or in various conventional breeding conditions. Animal models could also be used for analyzing the potential synergistic effect of different immunological trigger in the presence of bacterial products: this point has been indicated as “ecology of danger” as suggested by DA Clark (80). These studies are however not feasible to carry out in human subjects.

## Author Contributions

CA, AM, FB, GR, UK, and RB reviewed the literature and wrote sections of the review article. CA and RB created figures. All authors contributed to manuscript revision and approved the submitted version.

### Conflict of Interest

The authors declare that the research was conducted in the absence of any commercial or financial relationships that could be construed as a potential conflict of interest.
